# Multimorbidity and chronic diseases among undocumented migrants: evidence to contradict the myths

**DOI:** 10.1186/s12939-020-01225-0

**Published:** 2020-07-06

**Authors:** Luis Andrés Gimeno-Feliu, Marta Pastor-Sanz, Beatriz Poblador-Plou, Amaia Calderón-Larrañaga, Esperanza Díaz, Alexandra Prados-Torres

**Affiliations:** 1 EpiChron Research Group on Chronic Diseases, Aragón Health Sciences Institute (IACS), IIS Aragón, Miguel Servet University Hospital, Zaragoza, Spain; 2 Aragón Healthcare Service, San Pablo Health Centre, Zaragoza, Spain; 3 Health Services Research on Chronic Patients Network (REDISSEC), Carlos III Health Institute, Madrid, Spain; 4 Department of Medicine, Psychiatry and Dermatology, University of Zaragoza, Zaragoza, Spain; 5 Aragón Healthcare Service, Utrillas Health Centre, Zaragoza, Spain; 6 Department of Neurobiology, Care Sciences and Society, Aging Research Center, Karolinska Institutet, Stockholm, Sweden; 7 Department of Global Public Health and Primary Care, Research Group for General Practice, University of Bergen, Bergen, Norway; 8 Norwegian Centre for Minority Health Research, Oslo, Norway

**Keywords:** Emigration and immigration, Primary health care, Multimorbidity, Chronic disease, Spain, Health status

## Abstract

**Background:**

There is little verified information on the global health status of undocumented migrants (UMs). Our aim is to compare the prevalence of the main chronic diseases and of multimorbidity in undocumented migrants, documented migrants, and Spanish nationals in a Spanish autonomous community.

**Methods:**

Retrospective observational study of all users of the public health system of the region of Aragon over 1 year (2011): 930,131 Spanish nationals; 123,432 documented migrants (DMs); and 17,152 UMs. Binary logistic regression was performed to examine the association between migrant status (Spanish nationals versus DMs and UMs) and both multimorbidity and individual chronic diseases, adjusting for age and sex.

**Results:**

The prevalence of individual chronic diseases in UMs was lower than in DMs and much lower than in Spanish nationals. Comparison with the corresponding group of Spanish nationals revealed odds ratios (OR) of 0.1–0.3 and 0.3–0.5 for male and female UMs, respectively (*p* < 0.05 in all cases). The risk of multimorbidity was lower for UMs than DMs, both for men (OR, 0.12; 95%CI 0.11–0.13 versus OR, 0.53; 95%CI 0.51–0.54) and women (OR, 0.18; 95%CI 0.16–0.20 versus OR, 0.74; 95%CI 0.72–0.75).

**Conclusions:**

Analysis of data from a health system that offers universal coverage to all immigrants, irrespective of legal status, reveals that the prevalence of chronic disease and multimorbidity is lower in UMs as compared with both DMs and Spanish nationals. These findings refute previous claims that the morbidity burden in UM populations is higher than that of the native population of the host country.

## Background

Migration is a universal phenomenon. According to the 2018 United Nations Migration Report, the number of migrants worldwide reached 244 million in 2015 and is expected to increase further [1]. Recent years have seen growing interest in the health status of migrants, their impact on the health systems of host countries, and the best way to provide health care in accordance with human rights obligations [2–7].

A small but noteworthy portion of the general migrant population consists of migrants without legal authorization to reside in the host country. These people are referred to as undocumented migrants (UMs) or migrants in an irregular situation. UMs include visa “overstayers”, those who have lost resident status, rejected asylum seekers, and individuals who have entered a country illegally [8]. During the period 2002–2008, an estimated 1.9–3.8 million UMs were living in the 27 countries of the European Union [8]. In recent years, several countries have restricted the access of this population to public health care to prevent supposed “health tourism”, arguing that migrants migrate to host countries for treatment of pre-existing medical conditions [6].

Numerous studies of the general migrant population have confirmed the “healthy migrant effect”, and show that within this population health status is better at arrival but rapidly declines with increased length of stay in the host country [9–12]. However, owing to great inconsistency in the demographic and health data of migrants, there is a major knowledge gap regarding the health status of UMs [8]. In many countries, these people have great difficulty accessing the public health system because their right to access public health care is not recognized [4]. Consequently, they typically access public health care sporadically, often only in cases of medical emergency. Few studies have conducted global assessments of the health status of migrant populations: many more have focused on specific conditions (e.g. infectious diseases or mental health). Moreover, much of the available research has been conducted by NGOs, often based on case series, data from clinics that specialize in a specific type of disease (e.g., HIV), or very specific groups of sick people. Most of these studies make no comparison with documented migrants (DMs) or with the native population of the host country. The global health status of UMs is thus very difficult to determine [8, 13].

Two variables can potentially exert opposing influences on the health status of this group. On the one hand, the health of UMs may be worse than that of other immigrants given the greater degree of social exclusion of the former group. However, it is also possible that the “healthy migrant effect” is stronger in the case of UMs: in this group, health capital may be more necessary for successful migration, resulting in greater selection for healthy migrants before migration occurs.

The Spanish National Health System provides universal coverage and is almost fully funded by taxes. Care provision is free of charge at the point of delivery, resulting in a practically free system. Primary care centers serve as gatekeepers and are distributed to guarantee appropriate geographical coverage [14]. From 2000 to 2012, immigrants were guaranteed legal access to the same health care services as Spanish nationals, regardless of legal status [15]. In 2012, a central government decree withdrew this right, invalidating the health cards of UMs [5, 15]. According to reports by the Spanish government, this policy affected 870,000 UMs [15].

This legislative reform took place in a context where preventing “health tourism” by immigrants was pinpointed as a priority by the central government. This initiative was contested by the civil population, NGOs and scientific societies (such as the Spanish Society of Family and Community Medicine), who challenged these myths and called for civil disobedience against this law. In Aragon, the regional government through a local regulation reinstated the right of undocumented migrants to own a health card [16].

Between 2000 and 2012, in Spain there was no distinction in terms of public health care access for UMs, DMs, and Spanish nationals. Consequently, analysis of the prevalence of chronic diseases and multimorbidity [17] (the simultaneous presence of 2 or more chronic diseases) using data from this period provides us with a comprehensive view of the health status of this population.

The aim of this study is to characterize chronic diseases and multimorbidity according to migrant status (Spanish nationals versus DMs and UMs) using data from a universal coverage health system accessible to all inhabitants irrespective of legal status.

## Methods

This was a retrospective observational study based on the EpiChron Cohort, which gathers clinical and administrative data at the individual level from electronic health records (EHRs) and the health insurance database for almost all inhabitants of Aragon (approximately 98% of the total number of inhabitants in the region) [17]. The Aragon Health Service is part of the Spanish National Health System. In 2014, immigrants in Aragon accounted for 12.7% of the population and migrated to Spain primarily for economic reasons [18].

For each patient aged 18 years and older, demographic variables including age, sex, country of birth, and length of residence in Aragon were extracted from the health insurance database for the year 2011. Diagnoses were obtained from primary care EHRs, coded according to the International Classification of Primary Care (ICPC-2). Subsequently, ICPC-2 codes were grouped in expanded diagnostic clusters (EDCs) using the Adjusted Clinical Groups (ACG) System (version 10) [19]. The 114 chronic EDCs (from a total of 264 EDCs) included in the study were selected based on the list published by Salisbury et al. in 2011 [20]. Those authors defined a chronic disease as one lasting 6 months or more, including past conditions that required continuing care, major diseases with a risk of recurrence, and/or past diseases with continued implications for patient management. A dichotomous variable named multimorbidity (yes/no), defined as the presence of 2 or more distinct chronic EDCs, was created based on the total number of chronic EDCs assigned to each person. The term migrant was defined as any foreign-born person, regardless of nationality and duration of residence in Spain [21]. A UM was defined as any individual whose health card was invalidated as of September 1^st^ 2012 as a result of RDL16 / 2012, which removed the right to public health care access from non-nationals without a valid residence permit [15].

Binary logistic regression was performed to study the association between migrant status (Spanish nationals versus DMs and UMs) and both multimorbidity and individual chronic diseases, adjusted by age (as a categorical variable) and stratified by sex, length of stay, and area of birth. In a sensitivity analysis, all analyses were further adjusted by the number of visits to primary care, in order to account for potential disease under-diagnosis or under-registration due to lack of engagement with health services. All analyses were performed by grouping the study population as Spanish nationals, DMs, and UMs, and were repeated after stratifying the migrant population according to area of origin (Africa, Asia, Eastern Europe, Latin America, and Western Europe & North America). The Spanish national population served as the reference group. Statistical analyses were performed using STATA (version 12; StataCorp, College Station, TX, USA). The study was approved by the Ethics Committee for Clinical Investigation of Aragon.

## Results

We analyzed data from 1,070,715 individuals: 930,131 Spanish nationals, 123,432 DMs, and 17,152 UMs (Table 1). The distribution of the migrant population according to geographic area of origin is shown in Supplementary Table 1. Table 2 shows the overall prevalence of the 10 most frequent chronic diseases as well as the prevalence of multimorbidity.

**Table 1:** Demographic characteristics of the study population

	Spanish-nationals	Foreign-born (all)		Africa		Asia		Eastern Europe		Latin America		Western Europe & North America	
		DM	UM	DM	UM	DM	UM	DM	UM	DM	UM	DM	UM
**N**	930,131	123,432	17,152	29,643	2563	4621	327	39,678	7429	39,829	4643	9661	2190
**Men, %**	48.58	51.45	66.24	68.04	81.23	56.76	62.39	48.77	67.88	40.91	54.47	52.51	68.63
**Women, %**	51.42	48.55	33.76	31.96	18.77	43.24	37.61	51.23	32.12	59.09	45.53	47.49	31.37
**Age**													
**Mean (SD)**	51.90 (0.02)	38.18 (0.034)	38.06 (0.083)	37.39 (0.063)	37.85 (0.204)	38.49 (0.184)	39.71 (0.664)	36.71 (0.053)	37.23 (0.118)	38.51 (0.063)	38.89 (0.170)	45.12 (0.147)	39.16 (0.248)
**18–44 years, %**	39.71	74.11	76.10	78.27	80.53	72.80	69.11	78.79	78.58	71.39	72.73	53.97	70.68
**45–64 years, %**	32.14	22.94	22.11	19.48	17.17	23.31	26.91	20.26	20.62	25.14	24.6	35.26	26.94
**65+ years, %**	28.16	2.95	1.79	2.25	2.3	3.90	3.98	0.95	0.79	3.47	2.67	10.78	2.37
**Number of chronic diseases**													
**Mean (SD)**	2.01 (0.0025)	0.81 (0.0037)	0.26 (0.0055)	0.67 (0.0067)	0.24 (0.0133)	0.56 (0.0169)	0.24 (0.0403)	0.651 (0.0055)	0.20 (0.0072)	1.00 (0.0072)	0.36 (0.0124)	1.25 (0.0184)	0.25 (0.0156)
**0 diseases, %**	34.19	57.59	83.34	61.80	83.30	68.88	86.54	62.59	86.57	50.41	77.51	48.33	84.29
**1 disease, %**	20.39	22.62	11.13	22.61	12.72	18.35	7.95	21.57	9.17	24.31	14.69	22.00	10.46
**≥ 2 diseases, %**	45.42	19.79	5.53	15.59	3.98	12.77	5.51	15.84	4.26	25.28	7.80	29.67	5.25
**Duration of residence in Aragon**													
**< 5 years, %**	─	31.39	46.18	32.37	40.11	42.29	44.34	36.04	51.92	28.68	39.84	15.27	47.53
**≥ 5 years, %**	─	68.61	53.82	67.63	59.89	57.71	55.66	63.96	48.08	71.32	60.16	84.73	52.47
**Number ofvisits to the primary care doctor (normal care)**													
**Mean (SD)**	6.7 (8.4)	4.0 (5.6)	0.5 (2.0)	4.1 (5.6)	0.8 (2.2)	2.7 (4.6)	0.2 (0.8)	3.3 (5.1)	0.5 (1.9)	4.5 (5.8)	0.6 (2.2)	4.5 (6.6)	0.3 (1.8)

*N* number of persons, *DM* documented migrants, *UM* undocumented migrants

**Table 2:** Overall prevalence of the 10 most frequent chronic diseases and of multimorbidity among Spanish nationals, documented migrants, and undocumented migrants

Men				Women			
	Spanish nationals	DM	UM		Spanish nationals	DM	UM
Multimorbidity	39.58%	13.93%	3.69%	Multimorbidity	50.94%	26.01%	8.55%
Hypertension	22.09%	5.34%	2.10%	Hypertension	24.83%	6.43%	3.19%
Dyslipidemia	20.84%	9.23%	2.96%	Dyslipidemia	20.58%	8.19%	3.09%
Diabetes	8.66%	2.56%	1.07%	Varicose legs	15,43%	9,37%	3,64%
Arthropathy	7.72%	1.68%	0.53%	Artropathy	14.20%	3.32%	1.28%
Dermatitis	6.37%	4.70%	1.09%	Depression	12.13%	5.34%	2.18%
Obesity	6.16%	2.37%	0.81%	Thyroid disease	10.00%	5.69%	2.42%
Prostate hypertrophy	6.38%	0.58%	0.12%	Osteoporosis	10.32%	1.60%	0.57%
Depression	4.58%	1.60%	0.64%	Obesity	8.62%	5.38%	2.24%
Low back pain	3.75%	4.36%	0.52%	Dermatitis	7.57%	6.90%	1.61%
COPD	4.28%	0.56%	0.20%	Diabetes	7.35%	2.51%	1.23%

*DM* documented migrants, *UM* undocumented migrants

**Fig. 1:**
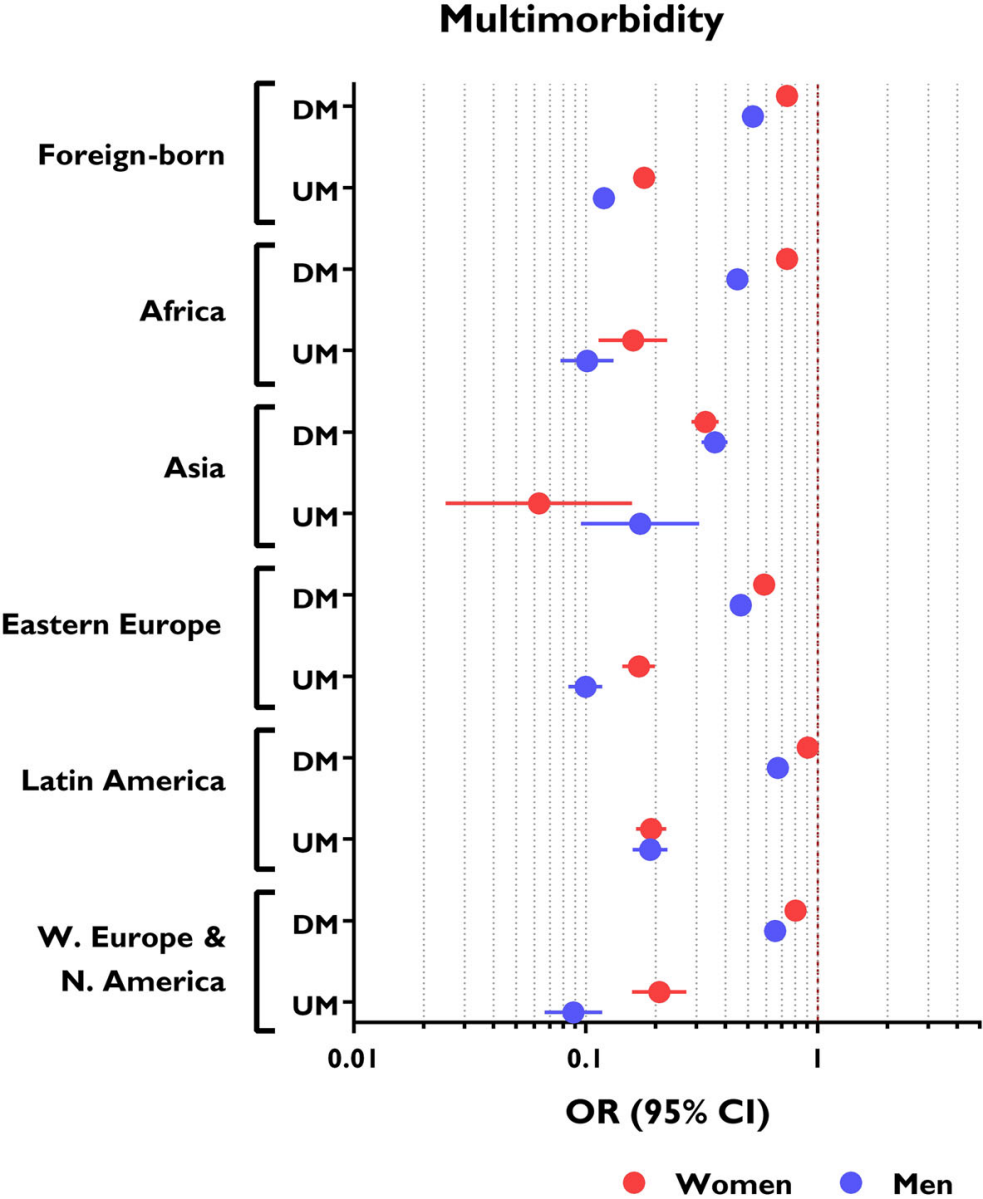
Risk of multimorbidity in the migrant population, adjusted for age and stratified by area of origin, legal status in Spain, and sex. Reference group: Spanish nationals. OR, odds ratio; DM, documented migrants; UM, undocumented migrants

The prevalence of multimorbidity was lower in migrants than in Spanish nationals. The risk of multimorbidity was lower in UMs and in DMs, both for men (OR, 0.12; 95%CI 0.11–0.13 and OR, 0.53; 95%CI 0.51–0.54, respectively) and women (OR, 0.18; 95%CI 0.16–0.20 and OR, 0.74; 95%CI 0.72–0.75, respectively) (Fig. 1).

Among UMs, the prevalence of multimorbidity (adjusted by age) was higher in women (11.82%; 95%CI 10.27%–13.37%) than men (8.45%; 95%CI 6.92%–9.97%). This difference was also observed in DMs versus Spanish nationals. Stratification according to area of origin had no effect on the prevalence of multimorbidity in UMs.

In UMs, the prevalence of multimorbidity (adjusted by age) was independent of length of stay: 9.74% (95%CI 8.54%–10.94%) in those with a length of stay ≥  5 years and 12.27% (95%CI 9.85%–14.68%) in those with a length of stay < 5 years. By contrast, in the DM population, length of stay was associated with a higher prevalence of multimorbidity: 28.13% (95%CI 27.74%–28.52%) in those with a length of stay ≥ 5 years versus 17.16% (95%CI 16.22%–18.11%) in those with a length of stay < 5 years.

**Fig. 2:**
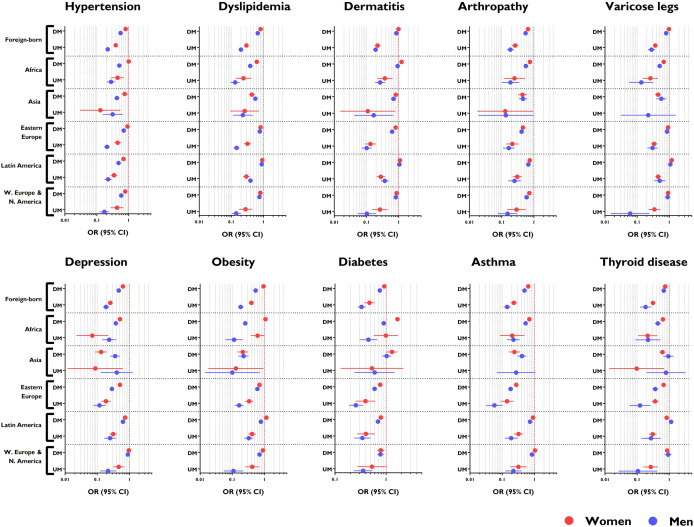
Risk of individual chronic diseases in the migrant population, adjusted for age and stratified by area of origin, legal status in Spain, and sex. Reference group: Spanish nationals. OR, odds ratio; DM, documented migrants; UM, undocumented migrants

The prevalence of chronic diseases was much lower in UMs versus DMs, and was always lower in UMs versus Spanish nationals, as shown in Table 2. In general, the OR ranged from 0.1–0.3 in male UMs and 0.3–0.5 in female UMs (Fig. 2).

A sensitivity analysis in which models were further adjusted by the number of visits to primary care revealed no significant changes with respect to the aforementioned findings (data not shown).

## Discussion

### Summary

This study, which compares the prevalence of chronic diseases and multimorbidity among Spanish nationals, DMs, and UMs using data from a health system in which all 3 groups were equally and universally covered, includes one of the largest sample sizes of UMs studied to date. The prevalence of chronic diseases was lower in UMs than in both DMs and Spanish nationals, and in DMs than in Spanish nationals. The prevalence of multimorbidity was lower in UMs than in DMs and in Spanish nationals, and increased with length of stay only in the DM group. These findings do not support claims of a higher morbidity burden in UMs and of health tourism as a driver of migration [6].

### Strengths and limitations

The main strength of this study is its large scale: all UMs for whom real-world data from EHR were available were included in the study. Other strengths include the region-wide coverage, the inclusion of all registered immigrants, and the lack of selection bias. The data analyzed are derived from the EpiChron Cohort, and have been analyzed in several similar studies in recent years [10, 17, 19, 22]. At the time of the study, UMs had unrestricted access to the public health system, regardless of their legal status. The measurement of individual-level morbidity burden using an internationally validated tool and data from EHR ensures a broad and reliable assessment of the health status of the migrant population [23]. Our study was based on diagnoses made by physicians, avoiding self-reporting bias. Importantly, the global patterns and the prevalence of the most common chronic diseases were similar to those reported in previous studies [22, 24, 25]. To reduce potential misclassification of diagnoses by physicians, we used the EDCs created by the ACG system and selected those chronic diseases included in the list of Salisbury et al. [20], in accordance with previous multimorbidity studies [25]. Finally, the use of administrative data allowed us to study the effect of important socio-demographic factors, including area of origin and length of stay in the host country.

Several limitations of our study should be noted. We did not consider socio-economic variables such as income or education level. This personal information is not recorded in Spanish health care databases, and could not be obtained in any other way while preserving anonymity. Inclusion of these parameters in our analysis could have helped to account for some of the complex factors that condition the use of health care services, such as income, educational level, unemployment, housing, social class or working conditions [7, 26, 27]. Another limitation of our study relates to our inability to refute the “salmon bias” hypothesis or “unhealthy remigration effect”. This hypothesis proposes that severely ill migrants tend to return to their country of origin to be cared for by their families. Consequently, multimorbidity rates among immigrants may be underestimated. Although we cannot reject this possibility based on our findings, a recently published study appears to rule out this hypothesis as the main explanation for better health outcomes in migrant versus native populations [28]. Finally, although all immigrants have the right to request a health card regardless of their legal status (provided that they are registered in the local population census), it is possible that some may not apply for a health card unless they actually become ill. This would result in an overestimation of the morbidity burden in UMs compared with DMs and the native population, since healthy UMs would not be included in the administrative databases. Regardless, this bias would likely result in underestimation of the differences in the prevalence of chronic diseases and multimorbidity reported in the present study. A final limitation is the underrepresentation in the data source used of some subgroups of the migrant population, such as those born in Asia.

### Comparison with existing literature

To the best of our knowledge, only one study has previously assessed the prevalence of multimorbidity in UMs. This study of UMs treated in primary care in Switzerland [29] reported a prevalence of multimorbidity of 23% in women and 14% in men, as compared with corresponding values of 12% and 8% in the present study. One possible explanation for these contrasting findings is that our analysis included all individuals with a health card, regardless of whether they availed of public health care. By contrast, one of the inclusion criteria in the Swiss study was having attended a medical consultation during the year of the study. Other possible reasons for these divergent results are the older mean age of the population in the Swiss study (42 years versus 38 years in the present study), differences between the migrant groups studied, and differences in the diagnostic criteria applied between one country and another. However, the main limitation of the Swiss study was the lack of any comparison with Swiss nationals. A study of Spanish workers [30] reported a higher prevalence of poor health in UMs that had been in the country for more than 3 years than in Spanish nationals, but reported no differences between UMs according to length of stay. A Dutch study [31] of primary care consultations reported a much higher prevalence of diabetes in UMs than that found in our study (7.4% and 1.1%, respectively). The remaining articles published on the subject mainly consist of analyses of cases recorded in NGO clinics, and therefore suffer from significant selection biases. We have found no studies that have analyzed the health of UMs in the context of health systems that afford equal access to UMs, DMs, and nationals.

The lower prevalence of chronic diseases in UMs than DMs is an important finding. It is possible that among UMs, health capital is an even more important determinant of the ability to migrate. Without work or residence permits, UMs are obliged to work in the submerged economy, without access to many of the benefits offered by the welfare state. This type of migrant therefore fits well with the “healthy migrant” paradigm. Another possibility is that, even when sick, UMs do not engage with the public health system for fear of being identified and deported, even though professionals working in the Spanish health system are not obliged to provide police with any information about the legal status of their patients. Even in countries where UMs are fully entitled to care, informal barriers such as language and communication problems, transport problems, poor knowledge of the health care system, the lack of a social network, and fear of deportation can undermine accessibility [13]. This is in line with the Tudor Hart inverse care law: “*the availability of good medical care tends to vary inversely with the need for it in the population served*” [26].

We found that the prevalence of multimorbidity among UMs was not influenced by length of stay in the host country. This is not the case within the general migrant population [19]. The irregular situation of this population may prevent them from engaging with the public health system, potentially resulting in under-registration and inadequate follow-up of medical conditions. However, a 2002 Spanish study of 380 Ecuadorian immigrants reported no differences between UMs and DMs in terms of health system access [32]. The fact that length of stay does affect the burden of morbidity among DMs is consistent with the results of a recent study that also analyzed data from the EpiChron cohort, and found that mortality (a variable for which there is no under-registration in Spain) was very low in immigrants with a length of stay < 5 years but tended to increase with a longer length of stay [10].

## Conclusions

Our analysis of data from the Spanish public health system, which offered universal coverage to all immigrants at the time of the study irrespective of legal status, demonstrates that the prevalence of multimorbidity and chronic diseases in UMs is lower than that in DMs and Spanish nationals. These findings refute previous claims of a higher morbidity burden among UMs.

## Supplementary information

**Additional file 1: Supplementary Table 1.** Distribution of the migrant population according to geographic area of origin.

## Data Availability

Data are available upon reasonable request.
