# Supplementary material for: Multimorbidity and chronic diseases among undocumented migrants: evidence to contradict the myths
Source: Int J Equity Health. 2020 Jul 6;19:113. doi: 10.1186/s12939-020-01225-0 (PMC7336489; doi:10.1186/s12939-020-01225-0)
Supplement: Supplementary file 1 — Additional file 1: Supplementary Table 1. Distribution of the migrant population according to geographic area of origin. [file 12939_2020_1225_MOESM1_ESM.pdf]

Supplementary table 1- distribution of the migrant population according to geographic area of origin.

	DM	UM	Total
**Africa**	**29.643**	**2.563**	**32.206**
Morocco	14.683	977	15.660
Algeria	3.716	433	4.149
Senegal	2.749	223	2.972
Gambia	1.674	84	1.758
Ghana	1.502	145	1.647
Mali	1.192	91	1.283
Equatorial Guinea	935	151	1.086
Guinea	612	68	680
Nigeria	568	93	661
Cape Verde	419	33	452
Mauritania	378	42	420
Cameroon	178	19	197
Egypt	135	28	163
Guinea-Bissau	119	38	157
Angola	74	23	97
Burkina Faso	85	8	93
Ivory Coast	77	16	93
Tunisia	71	10	81
Republic of the Congo	63	12	75
Western Sahara	66	1	67
Congo, Democratic Republic of	41	7	48
Niger	35	4	39
Sierra Leone	19	20	39
Liberia	29	6	35
Togo	28	5	33
South African Republic	29	1	30
Kenya	25	5	30
Mozambique	16	5	21
Gabon	16	1	17
Sudan	14	0	14
Mauritius	11	0	11
Benin	10	1	11
Central African Republic	10	0	10
Rwanda	10	0	10
Other countries	54	13	67

**Asia**	**4.621**	**327**	**4.948**
China	2.971	203	3.174
Pakistan	753	30	783
Syria	127	6	133
Armenia	97	7	104
India	78	24	102
Jordan	80	5	85
Lebanon	64	1	65
Philippines	55	3	58
Turkey	49	7	56
Bangladesh	48	0	48
Japan	35	12	47
Iran	28	5	33
Thailand	21	3	24
Laos	21	0	21
Israel	19	2	21
Korea, South	19	0	19
Korea, North	15	4	19
Kazakhstan	14	3	17
Uzbekistan	14	2	16
USSR	10	4	14
Indonesia	9	1	10
Other countries	94	5	99
**Eastern Europe**	**39.678**	**7.429**	**47.107**
Romania	31.760	5.374	37.134
Bulgaria	3.010	451	3.461
Poland	1.714	1.089	2.803
Ukraine	1.644	201	1.845
Russia	581	69	650
Moldova	266	38	304
Lithuania	227	59	286
Slovakia	87	60	147
Slovakia	70	11	81
Hungary	61	27	88
Albania	51	12	63
Bosnia and Herzegovina	45	4	49
Georgia	43	11	54
Latvia	32	7	39
Yugoslavia	31	2	33
Croatia	17	6	23
Serbia	14	2	16

Slovenia	10	5	15
Other countries	15	1	16
**Latin America**	**39.829**	**4.643**	**44.472**
Ecuador	11.465	1.583	13.048
Colombia	7.823	1.017	8.840
Dominican Republic	2.968	134	3.102
Argentina	2.825	411	3.236
Peru	2.821	280	3.101
Nicaragua	2.501	132	2.633
Brazil	1.850	331	2.181
Cuba	1.787	123	1.910
Venezuela	1.262	116	1.378
Bolivia	950	121	1.071
Chile	916	119	1.035
Uruguay	688	61	749
Honduras	652	42	694
Mexico	467	111	578
Paraguay	295	39	334
El Salvador	227	6	233
Guatemala	91	4	95
Panama	84	5	89
Costa Rica	51	1	52
Dominica	48	4	52
Puerto Rico	42	1	43
Other countries	16	2	18
**Western Europe & North America**	**9.661**	**2.190**	**11.851**
France	3.302	252	3.554
Portugal	1.995	1.292	3.287
Germany	1.178	113	1.291
Italy	661	204	865
Switzerland	568	17	585
United Kingdom	561	97	658
United States	371	27	398
Belgium	250	31	281
Netherlands	239	45	284
Czech Republic	87	42	129
Canada	82	6	88
Andorra	65	2	67
Australia	61	1	62
Ireland	50	15	65
Denmark	38	3	41
Sweden	36	15	51

Austria	32	4	36
Greece	28	12	40
Finland	19	3	22
Norway	14	4	18
Other countries	24	5	29

DM: documented migrants; UM: undocumented migrants
